# New indexes of body fat distribution and sex-specific risk of total and cause-specific mortality: a prospective cohort study

**DOI:** 10.1186/s12889-018-5350-8

**Published:** 2018-04-02

**Authors:** Susanne Rost, Dennis Freuer, Annette Peters, Barbara Thorand, Rolf Holle, Jakob Linseisen, Christa Meisinger

**Affiliations:** 1 Helmholtz Zentrum München, German Research Center for Environmental Health, Institute of Epidemiology, Neuherberg, Germany; 2 Helmholtz Zentrum München, German Research Center for Environmental Health, Institute of Health Economics and Health Care Management, Neuherberg, Germany; 3 Institute for Medical Informatics, Biometry and Epidemiology; LMU Munich, Munich, Germany; 4 Chair of Epidemiology, Ludwig-Maximilians-Universität München, UNIKA-T Augsburg, Neusässer Str. 47, 86156 Augsburg, Germany

**Keywords:** Anthropometric measures, All-cause mortality, Cardiovascular mortality, Cancer mortality, Body adiposity index, KORA, Obesity, Prospective study

## Abstract

**Background:**

A number of prior studies have examined the association between anthropometric measures and mortality, but studies investigating the sex-specific predictive value of novel anthropometric measures on mortality are scarce so far. Therefore, we investigated the sex-specific relevance of the new anthropometric measures body adiposity index (BAI) and waist to height ratio (WHtR) as well as the common measures body mass index (BMI), waist circumference (WC), and waist to hip ratio (WHR) for cause-specific mortality risk.

**Methods:**

The analysis was based on data from the German population based KORA (Cooperative Health Research in the Region of Augsburg) Augsburg cohort study. A total of 6670 men and 6637 women aged 25 to 74 years at baseline examination were included. During a mean follow-up period of 15.4 years, 2409 persons died. Via Cox proportional hazard regression, the associations between the different anthropometric measures and all cause-, cardiovascular disease (CVD)- and cancer mortality were assessed.

**Results:**

BMI, WC, and WHR were significantly associated with all-cause and CVD-mortality in both sexes. WC and WHR were particularly associated with higher all-cause and CVD-mortality risk in women, while in men especially WHtR and BAI were strongly related to these outcomes. Females with WC, WHtR, and WHR measures in the 4th quartile compared with women in the 2nd quartile had a higher risk of death from cancer. Contrary, men in the lowest quartile of WC and WHtR in comparison to men in the 2nd quartile had a significantly elevated cancer mortality risk. BAI was no risk predictor for all-cause and cause-specific mortality in women.

**Conclusions:**

Central obesity reflects higher all-cause and CVD-mortality risk particularly in women. BAI and WHtR seem to be valid as risk predictors for all-cause and especially CVD mortality in men but not women. There are marked sex-differences regarding cancer mortality risk for the different anthropometric measures.

**Electronic supplementary material:**

The online version of this article (10.1186/s12889-018-5350-8) contains supplementary material, which is available to authorized users.

## Background

It is well known that obesity is a risk factor for cardiovascular and metabolic diseases [1] and also for the development of certain cancer entities [2]. Furthermore, the association between body fat and mortality has been frequently discussed in a number of previous studies [3–9]. Body mass index (BMI) is a measure of obesity that is widely used, but it is not suited to differentiate between lean mass and fat mass [10, 11]. It is an important limitation that BMI does not consider the distribution of body fat, because nowadays it is well known that a number of diseases and also mortality are more closely related to visceral fatty tissue accumulation than overall body fatness [12–15]. Other measures of obesity have been developed and studied which consider body fat distribution to provide a better input concerning visceral or central obesity like waist circumference (WC), waist-to-hip ratio (WHR) and waist-to-height ratio (WHtR). Finally, in 2011 a new measure called “body adiposity index” (BAI) was introduced to estimate body fat percentage. Prior studies have already tried to find out the anthropometric measure which is the best predictor of total and cause-specific mortality [5, 16]. Study results were partially inconsistent regarding the role of BMI [8]. So far, European studies that investigated the association between values of novel anthropometric measures and cause-specific mortality in men and women are scarce. Thus, the aim of the present study was to determine which body fat measure was significantly predictive of all-cause-, CVD- and cancer mortality. To investigate whether there are sex-specific particularities regarding the associations, the analysis was conducted separately in men and women including a sample of the general German population.

## Methods

### Study population

Data from the Cooperative Health Research in the Region of Augsburg (KORA) study which is a prospective population-based cohort study in Southern Germany were used. In brief, four independent cross-sectional surveys were conducted in 1984/85 (Survey (S) 1), 1989/90 (S2), 1994/95 (S3) and 1999/2001 (S4); individuals aged 25 to 64 (S1) / 74 (S2-S3) years were included. For the present analysis, data from altogether 13,869 participants of S2, S3, and S4 were available. Data from 562 participants were excluded due to missing values in one or more of the variables required for the multivariable analyses. Thus, the final data set consisted of 6670 men and 6637 women. All subjects were prospectively followed until 2011, so the maximum observation time was 22 years. The study design, sampling method and data collection have been described in detail elsewhere [17]. The study was approved by the ethics committee of the Bavarian Medical Association (“Bayerische Landesärztekammer”) and performed in accordance with the Declaration of Helsinki. All individuals whose data were included in the analyses gave their written informed consent to participate in the study.

### Outcome definition

The endpoints considered in our study were overall mortality and cause-specific mortality (any CVD and cancer). Mortality was recorded by regularly checking the vital status of all study participants through the registration offices inside and outside the study region. Once the health certificates have been received from the local health departments, they were coded by a single trained person using the 9th revision of the International Classification of Diseases (ICD-9). The coding of the causes of death was as follows: CVD (ICD-9 390–459, 798), cancer (ICD-9 140–208), and deaths from any cause (ICD-9 001–999). The data collection and procedures are described in more detail elsewhere [18].

### Anthropometric measures

We analysed and compared five different anthropometric measures: Waist circumference was measured at the level midway between the lower rib margin and the iliac crest in standing position. Hip circumference was recorded at the maximum circumference over the buttocks. As further measures we computed the waist to hip ratio and the waist to height ratio. BMI was calculated as weight divided by height squared in kg/m^2^. The formula used for body adiposity index was: BAI = [(hip circumference (cm)/height (m)^1.5^) − 18] × 100 [19]. All anthropometric measures were assessed by trained medical staff.

### Confounding variables

Information on age, sex, sociodemographic characteristics and medical histories was assessed by standardized computer-aided interviews. Study participants provided information on physical activity during leisure time, on alcohol consumption and smoking status. The leisure time activity collected separately for summer and winter has been combined and categorized as active and inactive. A study participant was considered active if he/she reported a leisure time activity of at least 1 h per week in at least one season.

The amount of daily alcohol intake was divided into 3 categories: 0, 0.1 to 19.9, and 20.0 or more g/d for women; and 0, 0.1 to 39.9, and 40 or more g/d for men. Smokers were divided into current (occasional or regular), ex- and never smokers.

Education level was defined as high in case of final secondary school examination (Abitur or Fachabitur) and otherwise as low. The variable “survey” forms the basic examination in which the respective subjects participated (S2, S3, or S4). The data collection and procedures regarding standardized medical examinations are described in more detail elsewhere [17].

### Statistical methods

Baseline characteristics in the subgroups were given as means ± standard deviation (SD) or frequencies (%). For testing whether there were differences between surviving and deceased subjects the Wilcoxon signed-rank test as a nonparametric test was used. Correlations between anthropometric measures were evaluated with the Pearson correlation analysis. Cox proportional hazards models were used to estimate the Hazard ratios (HR) and 95% confidence intervals (95% CI) for the association between anthropometric measures and mortality. Proportional hazards assumptions as a precondition for Cox regression analysis were tested graphically and in case of uncertainty also with time-interaction terms. Subjects were divided into quartiles of the different anthropometric parameters, separately for men and women. Then HRs with 95% CIs for total mortality and also for CVD and cancer mortality were calculated. Limits of the generated quartiles are shown in Table 1. In the analysis using the anthropometric measures as categorized variables, the second quartile was always used as the reference category. In additional analyses, Cox proportional hazards models have been performed using the WHO cut-off values for BMI (18.5–24.99; 25–29.99; > = 30 kg/m^2^). In this analyses, persons with an extremely low BMI < 18.5 (*n* = 86) were excluded; as reference category the group with a BMI between 18.5 and 24.99 kg/m^2^ was used.

**Table 1:** Sex-specific limits of quartiles in anthropometric measures

	25th pctl	50th pctl	75 pctl
Men			
Waist circumference (cm)	89.00	95.50	102.00
Body mass index (kg/m^2^)	24.78	26.92	29.39
Waist/height ratio	0.51	0.55	0.59
Waist/hip ratio	0.88	0.93	0.97
Body adiposity index	24.76	26.92	29.30
Women			
Waist circumference (cm)	74.00	82.00	91.00
Body mass index (kg/m^2^)	22.83	25.84	29.58
Waist/height ratio	0.45	0.51	0.57
Waist/hip ratio	0.76	0.80	0.85
Body adiposity index	28.22	31.77	36.15

Interactions of the anthropometric measures with sex and age were tested. For the Cox regression model initially the following variables as potential confounders were considered: sex, age, survey, education level, alcohol intake, smoking status and physical activity. As they all showed significance in the full model, they were kept in. Traditional risk factors like blood pressure, blood lipid values and diabetes were considered as mediators and therefore not included in the model. For the sub-analyses regarding the outcomes cancer and CVD mortality we used the same adjustment variables as mentioned above. Because of the non-linearity of the associations, cubic smoothing splines were fitted to the estimated hazard ratios to show the relationship between different anthropometric measures and mortality. Confidence bands are given to visualize the uncertainty of the estimations. A *p*-value of < 0.05 was considered statistically significant. All statistical analyses were conducted with the software package SAS 9.2 and R 3.3.2.

## Results

### Baseline characteristics

Of the 13,307 included individuals, 2409 died until 2011. Of those, 1032 persons died due to CVD (ICD-9390–459, 798), and 791 due to cancer (ICD-9140–208). The mean observation time was 15.4 years (SD 5.0). Tables 2 and 3 present the baseline characteristics of men and women in the different mortality groups (total, CVD, and cancer mortality). Compared to women, men at baseline had a significantly higher education; they drank significantly more alcohol, were significantly more often smokers and had significantly higher blood pressure values (all *p*-values < 0.0001). The five anthropometric measures waist circumference (WC), body mass index (BMI), waist to hip ratio (WHR), waist to height ratio (WHtR) and body adiposity index (BAI) were all significantly higher in subjects who died during follow-up, except for men in the cancer group. Compared to the total- and the cancer mortality group, men and women with CVD deaths had the highest level of serum cholesterol concentrations, were less physically active and revealed the highest values in all anthropometric measures. Men and women who died from cancer had the highest alcohol intake and were more frequently current smokers. They also had the lowest values in all anthropometric measures (except BMI in women) compared to the other mortality groups.

**Table 2:** Baseline characteristics in men (mean ± SD for continuous variables, % for categorized variables) for the total sample, total and cause-specific mortality

	Total sample	Total mortality			CVD deaths		Cancer deaths	
Men	*n* = 6670	no (*n* = 5160)	yes (*n* = 1510)		*n* = 647		*n* = 484	
Survey (n; S2/S3/S4)	2381/2282/2007	1579/1787/1794	802/495/213		361/198/88		247/166/71	
		Mean ± SD or %	Mean ± SD or %	*p* value	Mean ± SD or %	*p* value	Mean ± SD or %	*p* value
Age (y)	49.71 ± 13.83	46.04 ± 12.986	62.24 ± 10.020	< 0.0001	63.64 ± 9.077	< 0.0001	61.43 ± 9.843	< 0.0001
Low/high education level	78.20/21.80	75.02/24.98	89.07/10.93	< 0.0001	89.64/10.36	< 0.0001	89.67/10.33	< 0.0001
Alkohol intake 0/0.1–399/> 39.9 g/d	18.11/29.61/52.28	17.33/31.40/51.28	20.79/23.51/55.70	< 0.0001	23.34/24.42/52.24	< 0.0001	16.32/22.93/60.74	0.0003
Total cholesterol (mg/dl)	233.25 ± 45.84	230.50 ± 44.998	242.66 ± 47.411	< 0.0001	248.30 ± 46.339	< 0.0001	237.98 ± 47.371	0.0064
Smoking status curr/ex/never	30.27/38.74/30.99	30.12/36.12/33.76	30.79/47.68/21.52	< 0.0001	26.89/50.39/22.72	< 0.0001	35.33/47.31/17.36	< 0.0001
Phys.act active/inactive	44.90/55.10	49.57/50.43	28.94/71.06	< 0.0001	26.89/73.11	0.0002	32.85/67.15	< 0.0001
Act. Hypertension y/n	45.72/54.28	40.00/60.00	65.23/34.77	< 0.0001	70.48/29.52	< 0.0001	58.47/41.53	< 0.0001
Blood pressure syst (mmHg)	135.1 ± 17.8	132.93 ± 16.40	142.64 ± 20.09	< 0.0001	145.14 ± 21.35	< 0.0001	140.81 ± 18.46	< 0.0001
Blood pressure diast (mmHg)	82.3 ± 11.0	82.53 ± 10.64	81.59 ± 12.00	< 0.0001	82.44 ± 13.29	< 0.0001	81.43 ± 10.62	< 0.0001
Weight (kg)	82.83 ± 12.31	83.05 ± 12.050	82.05 ± 13.154	0.0003	83.04 ± 13.176	0.8322	81.15 ± 12.26	0.0012
Waist circumference (cm)	95.99 ± 10.62	94.91 ± 10.356	99.65 ± 10.693	< 0.0001	100.74 ± 10.612	< 0.0001	98.41 ± 10.207	< 0.0001
Hip circumference (cm)	103.68 ± 7.06	103.47 ± 6.79	104.40 ± 7.865	0.0013	105.22 ± 7.944	< 0.0001	103.61 ± 7.102	0.9006
Body mass index (kg/m^2^)	27.29 ± 3.79	27.05 ± 3.673	28.12 ± 4.080	< 0.0001	28.55 ± 4.049	< 0.0001	27.68 ± 3.856	0.0101
Waist to height ratio	0.551 ± 0.066	0.542 ± 0.063	0.584 ± 0.065	< 0.0001	0.591 ± 0.064	< 0.0001	0.576 ± 0.063	< 0.0001
Waist to hip ratio	0.925 ± 0.064	0.916 ± 0.063	0.954 ± 0.060	< 0.0001	0.957 ± 0.059	< 0.0001	0.949 ± 0.060	< 0.0001
Body adiposity index	27.19 ± 3.72	26.70 ± 3.501	28.87 ± 3.932	< 0.0001	29.36 ± 3.924	< 0.0001	28.35 ± 3.726	< 0.0001

**Table 3:** Baseline characteristics in women (mean ± SD for continuous variables, % for categorized variables) for the total sample, total and cause-specific mortality

	Total sample	Total mortality			CVD deaths		Cancer deaths	
Women	*n* = 6637	No (*n* = 5738)	Yes (*n* = 899)		*n* = 385		*n* = 307	
Survey (n; S2/S3/S4)	2306/2230/2101	1798/1950/1990	508/280/111		224/126/35		172/79/56	
		Mean ± SD or %	Mean ± SD or%	*p* value	Mean ± SD or %	*p* value	Mean ± SD or%	*p* value
Age (y)	49.18 ± 13.83	46.92 ± 13.041	63.54 ± 9.381	< 0.0001	64.92 ± 8.283	< 0.0001	61.24 ± 10.193	< 0.0001
Low/high education level	86.61/13.39	85.27/14.73	95.11/4.89	< 0.0001	95.58/4.42	< 0.0001	92.51/7.49	0.0019
Alkohol intake 0/0.1–19.9/> 19.9 g/d	43.39/39.19/17.42	41.41/40.68/17.92	56.06/29.70/14.24	< 0.0001	57.66/27.79/14.55	0.0002	51.79/32.90/15.31	0.0095
Total cholesterol (mg/dl)	231.09 ± 45.27	227.71 ± 43.850	252.65 ± 48.214	< 0.0001	259.36 ± 46.107	< 0.0001	244.96 ± 48.43	< 0.0001
Smoking status current/ex/never	21.29/20.25/58.46	21.87/20.98/57.15	17.58/15.57/66.85	< 0.0001	15.06/15.58/69.35	< 0.0001	19.54/15.31/65.15	< 0.0001
phys.act active/inactive	41.45/58.55	44.14/55.86	24.25/75.75	< 0.0001	21.56/78.44	< 0.0001	28.01/71.99	< 0.0001
Hypertension yes/no	33.39/66.61	28.81/71.19	62.63/37.37	< 0.0001	70.13/29.87	< 0.0001	55.05/44.95	< 0.0001
Blood pressure systolic (mmHg)	127.24 ± 20.06	124.88 ± 18.70	142.34 ± 21.82	< 0.0001	145.28 ± 22.38	< 0.0001	139.01 ± 21.49	< 0.0001
Blood pressure diastolic (mmHg)	78.02 ± 10.65	77.76 ± 10.44	79.68 ± 11.77	< 0.0001	80.09 ± 12.30	< 0.0001	79.33 ± 11.23	< 0.0001
Weight (kg)	69.24 ± 12.99	68.91 ± 12.937	71.39 ± 13.158	< 0.0001	71.62 ± 13.555	< 0.0001	72.13 ± 13.029	< 0.0001
Waist circumference (cm)	83.36 ± 12.24	82.400 ± 12.044	89.47 ± 11.712	< 0.0001	89.94 ± 11.886	< 0.0001	89.32 ± 11.482	< 0.0001
Hip circumference (cm)	103.37 ± 10.55	102.95 ± 10.494	106.03 ± 10.530	< 0.0001	106.50 ± 10.584	< 0.0001	106.0 ± 10.448	< 0.0001
Body mass index (kg/m^2^)	26.62 ± 5.09	26.33 ± 5.035	28.50 ± 5.016	< 0.0001	28.69 ± 5.083	< 0.0001	28.52 ± 5.17	< 0.0001
Waist to height ratio	0.518 ± 0.081	0.510 ± 0.080	0.566 ± 0.077	< 0.0001	0.570 ± 0.077	< 0.0001	0.562 ± 0.078	< 0.0001
Waist to hip ratio	0.805 ± 0.064	0.800 ± 0.062	0.843 ± 0.058	< 0.0001	0.843 ± 0.059	< 0.0001	0.840 ± 0.059	< 0.0001
Body adiposity index	32.549 ± 6.067	32.11 ± 5.971	35.38 ± 5.918	< 0.0001	35.75 ± 5.769	< 0.0001	35.07 ± 6.158	< 0.0001

The calculated correlation coefficients between the anthropometric measures showed high correlation coefficients, in particular between BMI and BAI, WC, and WHtR (Table 4).

**Table 4:** Correlation matrix of the anthropometric measures (total sample)

	BMI	BAI	WC	WHR	WHtR
BMI	1.000	0.716	0.808	0.440	0.876
BAI	0.716	1.000	0.354	−0.077	0.633
WC	0.808	0.354	1.000	0.820	0.924
WHR	0.440	−0.077	0.820	1.000	0.710
WHtR	0.876	0.633	0.924	0.710	1.000

### Anthropometric measures and all-cause mortality

Due to significant interactions with sex in the fully adjusted model all analyses were stratified by sex. The *P* values for interactions between sex and BMI, BAI, WC, WHR, and WHtR were 0.0662, 0.2669, 0.0002, 0.0001, and 0.0054, respectively (for outcome total mortality).

In multivariable analysis men and women in the highest quartile of BMI compared to subjects in the second quartile had a significantly elevated risk to die from any cause. Men but not women in the bottom quartile of BMI had also a higher all-cause mortality risk compared to the second quartile. Men in the fourth quartile of BAI, WC, and WHR and men in the first, third and fourth quartile of WHtR showed a significantly higher all-cause mortality risk in comparison to the reference group. However, in women a somewhat different association became apparent: there was no significant relationship of BAI and all-cause mortality. Women in the fourth quartile of WHtR and women in the third and fourth quartile of WC compared to the second quartile showed a significantly increased total mortality risk. Regarding WHR, women in the third and fourth quartile had a strongly elevated risk to die from all-cause in comparison to the reference group, whereas the first quartile had a lower risk (Table 5).

**Table 5:** Hazard ratios (HR) and confidence intervals (CI) for the association between the different anthropometric measures and total mortality for men and women by quartiles; the second quartile was set as the reference category

Total mortality	Men				Women			
	HR	95% CI		*p*-value	HR	95% CI		*p*-value
Body mass index								
1st quartile	1.18	1.00	1.38	0.044	1.12	0.88	1.43	0.347
2nd quartile	1.00				1.00			
3rd quartile	0.91	0.79	1.06	0.213	1.00	0.83	1.21	0.988
4th quartile	1.21	1.06	1.39	0.007	1.29	1.07	1.56	0.007
Body adiposity index								
1st quartile	1.12	0.93	1.34	0.250	1.09	0.84	1.42	0.520
2nd quartile	1.00				1.00			
3rd quartile	1.02	0.87	1.19	0.822	1.00	0.82	1.21	0.987
4th quartile	1.23	1.06	1.42	0.006	1.18	0.98	1.43	0.085
Waist circumference								
1st quartile	1.15	0.97	1.37	0.118	0.98	0.74	1.31	0.904
2nd quartile	1.00				1.00			
3rd quartile	0.99	0.85	1.15	0.909	1.22	1.01	1.49	0.043
4th quartile	1.28	1.12	1.47	<.0001	1.63	1.35	1.97	<.0001
Waist to hip ratio								
1st quartile	1.04	0.85	1.26	0.717	0.68	0.50	0.93	0.015
2nd quartile	1.00				1.00			
3rd quartile	1.12	0.97	1.30	0.119	1.29	1.05	1.58	0.014
4th quartile	1.35	1.17	1.55	<.0001	1.63	1.35	1.97	<.0001
Waist to height ratio								
1st quartile	1.34	1.10	1.64	0.004	1.03	0.76	1.40	0.846
2nd quartile	1.00				1.00			
3rd quartile	1.16	1.00	1.35	0.054	1.20	0.98	1.47	0.079
4th quartile	1.46	1.27	1.69	<.0001	1.61	1.32	1.95	<.0001

Values are adjusted for age, survey, education level, alcohol intake, smoking status, physical activity, and time/smoking status interaction

### Anthropometric measures and CVD mortality

Regarding CVD mortality, men and women in the highest quartile of BMI, WHtR, and WHR had a significantly higher risk to die in comparison to persons in the second quartile (Table 6). While men in the third and fourth quartile of BAI had a significantly elevated CVD mortality risk compared to the reference group there was no association between BAI and CVD mortality in women. Increased WC at baseline also predicted CVD mortality in both sexes. Compared to the second quartile only men in the fourth quartile had a significantly elevated CVD mortality risk, while for women this was found in the third and fourth quartile.

**Table 6:** Hazard ratios (HR) and 95% confidence intervals (CI) for the association between the different anthropometric measures and cardiovascular mortality for men and women; the second quartile was set as the reference category

Cardiovascular mortality	Men				Women			
	HR	95% CI		*p*-value	HR	95% CI		*p*-value
Body mass index								
1st quartile	0.94	0.72	1.22	0.627	1.34	0.91	1.97	0.133
2nd quartile	1.00				1.00			
3rd quartile	0.92	0.74	1.16	0.493	1.16	0.86	1.56	0.339
4th quartile	1.30	1.06	1.61	0.013	1.46	1.09	1.97	0.012
Body adiposity index								
1st quartile	1.17	0.85	1.60	0.338	0.72	0.46	1.15	0.167
2nd quartile	1.00				1.00			
3rd quartile	1.29	1.01	1.66	0.043	0.83	0.62	1.12	0.227
4th quartile	1.54	1.22	1.96	<.0001	1.14	0.86	1.51	0.365
Waist circumference								
1st quartile	0.80	0.60	1.08	0.144	1.02	0.64	1.62	0.937
2nd quartile	1.00				1.00			
3rd quartile	0.97	0.77	1.21	0.778	1.36	1.01	1.85	0.045
4th quartile	1.28	1.04	1.57	0.018	1.70	1.27	2.28	<.0001
Waist to hip ratio								
1st quartile	0.86	0.63	1.18	0.348	0.79	0.50	1.25	0.309
2nd quartile	1.00				1.00			
3rd quartile	1.05	0.84	1.31	0.693	1.20	0.88	1.65	0.249
4th quartile	1.33	1.07	1.64	0.009	1.66	1.24	2.22	0.009
Waist to height ratio								
1st quartile	0.87	0.61	1.25	0.460	0.82	0.48	1.41	0.476
2nd quartile	1.00				1.00			
3rd quartile	1.14	0.90	1.44	0.280	1.23	0.90	1.68	0.196
4th quartile	1.56	1.25	1.94	<.0001	1.62	1.20	2.19	0.002

Values are adjusted for age, survey, education level, alcohol intake, smoking status, physical activity, and time/smoking status interaction

### Anthropometric measures and cancer mortality

The risk to die from cancer increased in women in the fourth quartile of WC, WHR, and WHtR in comparison to women in the second quartile. Furthermore, females in the lowest quartile of WHR had a significantly lower cancer mortality risk compared to the reference group. In women there was no association found between BMI as well as BAI and cancer mortality. A different picture appeared regarding the association between anthropometric measures and cancer mortality in men. Males in the bottom quartile of BMI, WC, and WHtR had a significantly higher risk to die from cancer in comparison to the reference group. There was no association between BAI as well as WHR and cancer mortality in men (Table 7).

**Table 7:** Hazard ratios (HR) and 95% confidence intervals (CI) for the association between the different anthropometric measures and cancer mortality for men and women; the second quartile was set as the reference category

Cancer mortality	Men				Women			
	HR	95% CI		*p*-value	HR	95% CI		*p*-value
Body mass index								
1st quartile	1.33	1.02	1.73	0.037	0.93	0.62	1.39	0.715
2nd quartile	1.00				1.00			
3rd quartile	0.86	0.67	1.12	0.270	0.85	0.62	1.19	0.347
4th quartile	1.06	0.83	1.36	0.628	1.33	0.97	1.81	0.072
Body adiposity index								
1st quartile	1.08	0.79	1.47	0.627	1.20	0.79	1.83	0.395
2nd quartile	1.00				1.00			
3rd quartile	0.93	0.71	1.20	0.564	1.14	0.81	1.61	0.445
4th quartile	1.02	0.79	1.31	0.903	1.37	0.98	1.91	0.064
Waist circumference								
1st quartile	1.46	1.09	1.95	0.010	0.81	0.50	1.31	0.392
2nd quartile	1.00				1.00			
3rd quartile	0.99	0.76	1.30	0.962	1.04	0.74	1.46	0.811
4th quartile	1.24	0.97	1.58	0.088	1.73	1.26	2.36	0.001
Waist to hip ratio								
1st quartile	1.25	0.91	1.71	0.172	0.57	0.35	0.94	0.028
2nd quartile	1.00				1.00			
3rd quartile	1.03	0.80	1.34	0.805	1.20	0.86	1.66	0.283
4th quartile	1.25	0.98	1.60	0.076	1.39	1.02	1.91	0.039
Waist to height ratio								
1st quartile	1.74	1.26	2.39	0.001	0.96	0.59	1.54	0.851
2nd quartile	1.00				1.00			
3rd quartile	1.14	0.87	1.48	0.336	1.03	0.73	1.45	0.876
4th quartile	1.25	0.97	1.62	0.084	1.69	1.22	2.34	0.002

Values are adjusted for age, survey, education level, alcohol intake, smoking status, physical activity, and time/smoking status interaction

**Fig. 1:**
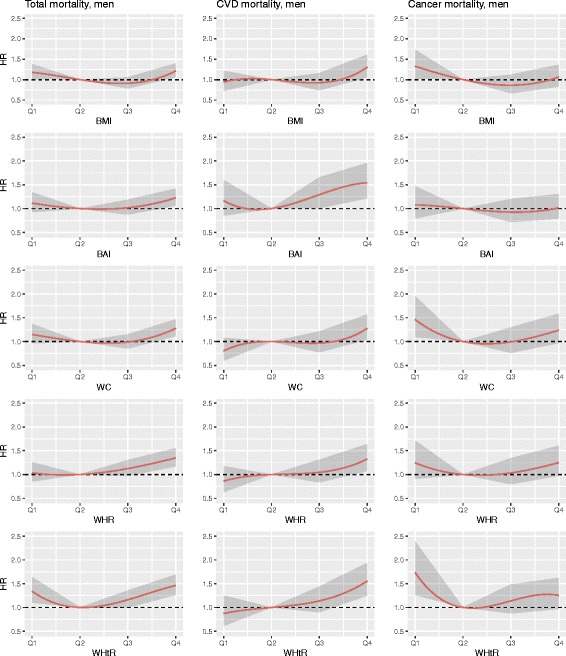
Association between BMI, BAI, WC, WHR, and WHtR and the outcomes all-cause-, CVD-, and cancer mortality using cubic smoothing splines in men

**Fig. 2:**
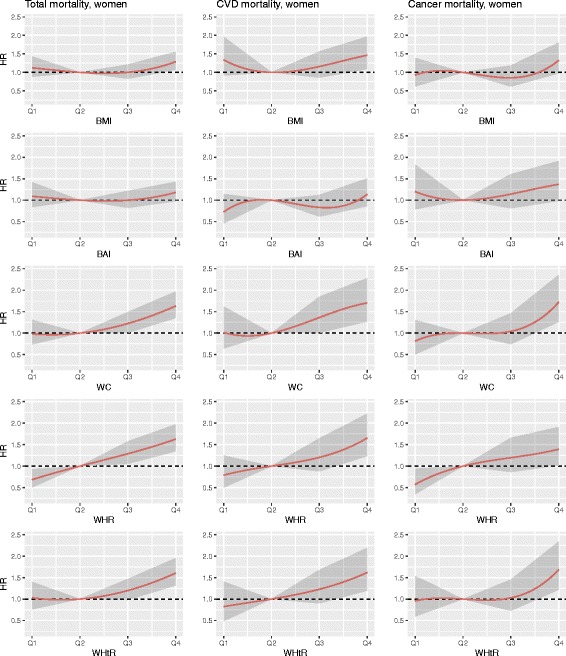
Association between BMI, BAI, WC, WHR, and WHtR and the outcomes all-cause-, CVD-, and cancer mortality using cubic smoothing splines in women

The association between the different anthropometric measures and the outcomes all-cause-, CVD-, and cancer mortality using cubic smoothing splines are shown in Fig. 1 (men) and Fig. 2 (women).

### Additional analyses

We also examined the association between BMI and cause-specific mortality using the WHO cut-off values (Additional file 1). It was found that men in the BMI group 25–29.99 kg/m^2^ had a significantly lower total and cancer mortality risk; only men with a BMI > = 30 kg/m^2^ had a significantly elevated CVD mortality risk. In women, however, there was a significantly increased total, CVD and cancer mortality risk in the category of a BMI > = 30 kg/m^2^.

Although there was no significant interaction with age, additionally age-stratified analysis (cut-off <=60/> 60 years) were conducted (see Additional file 2, Additional file 3, Additional file 4).

In the age-group > 60 years all 5 anthropometric measures were associated with total mortality; persons in the fourth quartile of the respective measure had an elevated mortality risk. Only persons in the bottom quartile of WHR compared to the second quartile had a lower total mortality risk. Regarding CVD mortality, BMI showed no association with the outcome; the over 60 years old persons in the bottom quartile of BAI, WC, WHR, and WHtR had a significantly lower CVD mortality risk. Furthermore, persons in the highest quartile of WHtR compared to the reference group had an elevated CVD mortality risk. Regarding cancer mortality only participants in the fourth quartile of BMI and WHtR compared to the reference category had a significantly elevated risk.

In the age-group <= 60 years, persons in the bottom quartile of all five measures had a significantly lower total mortality risk in comparison to the reference group. Furthermore, participants in the third and fourth quartile of all five measures (BMI only fourth quartile) had a significantly elevated total mortality risk in comparison to the second quartile; this was also the case with regard to CVD mortality. Compared to the reference group only persons in the bottom quartile of WHR and BAI had a significantly lower CVD mortality risk. Regarding cancer mortality, persons in the bottom quartile of BMI, WC, WHR, and WHtR had a significantly lower risk, while participants in the fourth quartile of BAI, WC, WHR, and WHtR had a significantly higher risk than the reference group.

## Discussion

### Key results

In this population-based cohort study, among the analysed anthropometric measures BMI, WC, and WHR were significantly associated with all-cause and CVD-mortality in men and women. Central obesity seems to reflect higher mortality risk particularly in women. In men, WHtR and BAI was found to be strongly and independently related to all-cause and CVD mortality, respectively. Regarding cancer mortality, a distinct mortality risk pattern turned out in men and women for the different anthropometric measures. The adiposity measure BAI may not play a role as risk predictor for all-cause and cause-specific mortality in women. All five measures were associated with the different mortality outcomes in persons <=60 as well as persons > 60 years, but the associations were more pronounced and stronger in the younger age-group.

### Body adiposity index as a risk predictor of mortality

The BAI has recently been proposed as an alternative index of obesity [19]. So far, prospective studies on the association between BAI as “novel” anthropometric measure and cause-specific mortality including men and women from the same population are scarce. In the present investigation, it was found that BAI was not a meaningful predictor for total or cause-specific mortality in women. To the best of our knowledge, no comparable population-based studies on this issue including women are available. Thus, this novel finding has to be confirmed or refuted in further prospective studies. A possible reason for our finding could be that BAI does not characterise central obesity, which is associated with higher mortality risk particularly in females. In addition, studies which investigated the validity of BAI to reflect body fat percentage found only a poor concordance [20, 21]. Contrary to women, in men, BAI turned out to be a good risk predictor particularly for CVD-mortality in the present study. Another investigation examining the association between BAI and all-cause as well as CVD-mortality including 19,756 adult men from Dallas, Texas, reported that BAI is not a better mortality predictor than BMI or WC [22]. Contrary to the present population-based study, that study included participants who were referred by their employers or physicians. Thus, the findings of the studies would not directly be comparable. An Australian study on 4175 males free of heart disease, diabetes and stroke from the population found that BAI may be of interest as a measure of obesity but that it was not an independent predictor of CVD and coronary heart disease mortality [23].

The found association between BAI and CVD mortality in men was comparable to the associations between the other investigated general obesity measure BMI and CVD mortality. Because BAI requires a mathematical calculation of some complexity, in clinical practice it seems easier to use the common anthropometric measures such as BMI to estimate someone’s CVD risk.

### Anthropometric measures and all-cause mortality

In the present study BMI showed a u-shaped association with all-cause mortality in men and women. This pattern is already well known, for example from a collaborative analysis of 57 prospective studies, where in both sexes mortality was lowest at a BMI about 22.5 to 25 kg/m^2^ [8]. In our study a significantly higher mortality risk from any cause in the lowest and highest quartiles of BMI were only shown in men. Waist to hip ratio showed a linear risk increase through all categories in women. In men, however, the rise was less distinct. In a meta-analysis from Czernichow et al. including nine UK cohort studies, they also found a significant risk increase for rising waist to hip ratios [9]. In that meta-analysis no testing for sex differences was reported. A Swedish study including female participants only showed that obesity does not seem to be a risk factor for increased mortality as long as it is not centrally located [24].

The present study showed a significantly elevated all-cause-mortality risk with increasing WHtR in men, a finding which corresponds to prior studies on this issue [16, 25].

### Anthropometric measures and CVD mortality

Central obesity as a risk factor for all-cause and CVD mortality has been described in a number of other studies [6, 7, 9]. In our study, central obesity was strongly associated with CVD mortality, particularly in women. A comparable study from the Netherlands that also looked for sex differences in all-cause and CVD mortality did not detect a sex difference in waist-associated measures [5]. However, they only considered subjects aged 55 and older, raising the question if there could be differences regarding the impact of central obesity on mortality risk in different age-groups. Further studies underlined that anthropometric measures of abdominal obesity were strongly and positively associated with all-cause and CVD-mortality in women [26, 27]. A high WHtR turned out as a significant risk predictor for CVD mortality in both sexes. This is in accordance with another study including 16,332 men in the Physicians’ Health Study and 32,700 women in the Women’s Health Study which demonstrated that WHtR was strongly associated with an increased CVD risk (including nonfatal myocardial infarction, nonfatal ischemic stroke, cardiovascular death) in men and women. Due to the somewhat different outcomes in the studies, the results are surely not entirely comparable [28].

### Anthropometric measures and cancer mortality

In the present study women with higher WC and WHtR had a significantly higher risk to die from cancer. Furthermore, females in the highest quartile of WHR in comparison to the reference group had a significantly elevated risk, whereas females in the first quartile had a lower risk. Contrary, in men lower values of BMI, WC, and WHtR were significantly related to higher cancer mortality. Previous studies predominantly looked for differences in the influence of BMI on cancer mortality. In addition they were mainly searching for differences between cancer sites and often did not focus on sex differences [2, 29]. From these studies it is known that mortality risk due to obesity differ strongly depending on the cancer sites. It is remarkable that overweight patients had a higher mortality risk mainly in hormone-associated cancers like postmenopausal breast cancer, endometrial and ovary cancers [29]. This provides us a possible explanation for the higher risk in women with central obesity, because their cause of death from cancer is more often an obesity-associated tumour. A study from Zhang et al. also reported a strongly positive association between abdominal obesity and cancer mortality in women [27]. Contrary, in men the higher cancer mortality in the bottom quartile in comparison to the reference group may be due for example to smoking-related tumours [30].

Other studies have also shown that a more android fat distribution in women, expressed as a higher WC, WHtR, and WHR, is associated with a higher risk of disease [26, 27, 31]. For females, measurements of the total fat mass of the body, such as BAI and BMI, might play a more subordinate role in terms of mortality risk. On the other hand, in men the general overweight and obesity measures BMI and BAI seems to be suitable as disease predictors in addition to the measures displaying fat distribution. BAI and BMI were highly correlated measures in our study sample and there was a clearer correlation between these measures and an increased overall and CVD mortality risk in men compared to women. As underlying mechanisms for the higher mortality risk associated with a visceral fat accumulation, particularly in women, a number of different causes are suggested [32, 33]. For example, visceral fat is metabolically active with secreting large quantities of proinflammatory cytokines, which could accelerate the atherosclerotic process and increase the risk of CVD [34]. The underlying mechanisms for the protective effect of gynoid and peripheral fat are unknown so far [35]. Furthermore, the mechanisms for the found differences between men and women are not entirely clear. More research is needed to elucidate what sex-specific mechanisms with regard to body fat distribution are involved in disease development.

### Strengths and limitations

The main strengths of the present study are the large sample size with a balanced proportion of men and women from a population based study and the long follow-up time of an average of 15 years with a high number of outcomes. In addition, all anthropometric measures were collected in a standardized way by trained staff, which is why we can expect reliable phenotyping. As a limitation, residual confounding by unmeasured variables for example dietary factors and comorbidities cannot be entirely ruled out. When evaluating the relation between anthropometric measures and mortality, it might be important to consider lifestyle factors such as diet. A healthier diet often in combination with a healthier overall lifestyle is associated with a lower risk of mortality and less overweight and obesity [36]. Thus, confounding by diet could have caused the independent effects found in our study to be overestimated. Furthermore, anthropometric measures were assessed at only one point in time, so the estimates could not account for changes in anthropometric measures during follow-up. This study included German subjects up to an age of 74 at baseline, thus, risks may differ in older people and persons of other ethnicity.

## Conclusions

In this population based cohort study well established anthropometric measures such as increased BMI, WC, WHtR, and WHR were significantly associated with a higher all-cause and CVD-mortality in men and women. Regarding the relationship between common anthropometric measures and cancer mortality, distinct sex-differences became apparent. The novel measure BAI seems to play a role as risk predictor for all-cause and particular CVD mortality in men only and to be not a useful measure in both sexes regarding the prediction of cancer mortality.

## Additional files

Additional file 1: BMI and cause-specific mortality. Hazard ratios (HR) and confidence intervals (CI) for the association between BMI and cause-specific mortality by WHO definition in men and women. (DOCX 14 kb)

Additional file 2: Different anthropometric measures and total mortality. Hazard ratios (HR) and confidence intervals (CI) for the association between the different anthropometric measures and total mortality for persons <=/> 60 years by quartiles; the second quartile was set as the reference category. (DOCX 21 kb)

Additional file 3: Different anthropometric measures and CVD mortality. Hazard ratios (HR) and confidence intervals (CI) for the association between the different anthropometric measures and CVD mortality for persons <=/> 60 years by quartiles; the second quartile was set as the reference category. (DOCX 21 kb)

Additional file 4: Different anthropometric measures and cancer mortality. Hazard ratios (HR) and confidence intervals (CI) for the association between the different anthropometric measures and cancer mortality for persons <=/> 60 years by quartiles; the second quartile was set as the reference category. (DOCX 21 kb)

## Abbreviations

BAI: Body adiposity index
BMI: Body mass index
CI: Confidence interval
CVD: Cardiovascular disease
HR: Hazard ratio
ICD: International Classification of Diseases
KORA: Cooperative Health Research in the Region of Augsburg
SD: Standard deviation
WC: Waist circumference
WHR: Waist to hip ratio
WHtR: Waist to height ratio
