# Supplementary material for: New indexes of body fat distribution and sex-specific risk of total and cause-specific mortality: a prospective cohort study
Source: BMC Public Health. 2018 Apr 2;18:427. doi: 10.1186/s12889-018-5350-8 (PMC5879745; doi:10.1186/s12889-018-5350-8)
Supplement: Supplementary file 1 — BMI and cause-specific mortality. Hazard ratios (HR) and confidence intervals (CI) for the association between BMI and cause-specific mortality by WHO definition in men and women. (DOCX 14 kb) [file 12889_2018_5350_MOESM1_ESM.docx]

Additional file 1: BMI and cause-specific mortality

Table Hazard ratios (HR) and confidence intervals (CI) for the association between BMI and cause-specific mortality by WHO definition in men and women

|  | **men** |  |  |  |  |  | **women** |  |  |  |
| --- | --- | --- | --- | --- | --- | --- | --- | --- | --- | --- |
|  | HR | 95% CI | | p-value | HR | 95% CI | |  | p-value |  |
| **Total mortality** |  |  |  |  |  |  |  |  |  |  |
| BMI 18.5-24.99 kg/m² | 1.00 |  |  |  | 1.00 |  |  |  |  |  |
| BMI 25.0-29.99 kg/m² | 0.84 | 0.73 | 0.96 | 0.009 | 1.06 | 0.89 | 1.26 |  | 0.527 |  |
| BMI >= 30 kg/m² | 1.08 | 0.93 | 1.26 | 0.293 | 1.00 | 0.83 | 1.21 |  | 0.001 |  |
|  |  |  |  |  |  |  |  |  |  |  |
| **CVD mortality** |  |  |  |  |  |  |  |  |  |  |
| BMI 18.5-24.99 kg/m² | 1.00 |  |  |  | 1.00 |  |  |  |  |  |
| BMI 25.0-29.99 kg/m² | 1.02 | 0.82 | 1.26 | 0.884 | 1.05 | 0.80 | 1.38 |  | 0.710 |  |
| BMI >= 30 kg/m² | 1.45 | 1.14 | 1.84 | 0.002 | 1.38 | 1.05 | 1.83 |  | 0.022 |  |
|  |  |  |  |  |  |  |  |  |  |  |
| **Cancer mortality** |  |  |  |  |  |  |  |  |  |  |
| BMI 18.5-24.99 kg/m² | 1.00 |  |  |  | 1.00 |  |  |  |  |  |
| BMI 25.0-29.99 kg/m² | 0.74 | 0.59 | 0.92 | 0.007 | 1.08 | 0.80 | 1.46 |  | 0.615 |  |
| BMI >= 30 kg/m² | 0.82 | 0.63 | 1.07 | 0.149 | 1.58 | 1.17 | 2.16 |  | 0.003 |  |

Values are adjusted for survey, education level, alcohol intake, smoking status, physical activity and time/smoking status interaction.
