# Supplementary material for: New indexes of body fat distribution and sex-specific risk of total and cause-specific mortality: a prospective cohort study
Source: BMC Public Health. 2018 Apr 2;18:427. doi: 10.1186/s12889-018-5350-8 (PMC5879745; doi:10.1186/s12889-018-5350-8)
Supplement: Supplementary file 2 — Different anthropometric measures and total mortality. Hazard ratios (HR) and confidence intervals (CI) for the association between the different anthropometric measures and total mortality for persons <=/> 60 years by quartiles; the second quartile was set as the reference category. (DOCX 21 kb) [file 12889_2018_5350_MOESM2_ESM.docx]

Additional file 2: Different anthropometric measures and total mortality

Table Hazard ratios (HR) and confidence intervals (CI) for the association between the different anthropometric measures and total mortality for persons <=/> 60 years by quartiles; the second quartile was set as the reference category.

q

| **Total mortality** |  | **<= 60 years** |  |  |  |  | **>60 years** |  |  |  |
| --- | --- | --- | --- | --- | --- | --- | --- | --- | --- | --- |
|  | HR | 95% CI | | p-value | HR | 95% CI | |  | p-value |  |
| **Body mass index** |  |  |  |  |  |  |  |  |  |  |
| 1st quartile | 0.74 | 0.58 | 0.94 | 0.016 | 1.06 | 0.93 | 1.22 |  | 0.392 |  |
| 2nd quartile | 1.00 |  |  |  | 1.00 |  |  |  |  |  |
| 3rd quartile | 0.94 | 0.76 | 1.16 | 0.541 | 1.01 | 0.88 | 1.16 |  | 0.894 |  |
| 4th quartile | 1.57 | 1.29 | 1.90 | <.0001 | 1.19 | 1.03 | 1.36 |  | 0.015 |  |
| **Body adiposity index** |  |  |  |  |  |  |  |  |  |  |
| 1st quartile | 0.73 | 0.59 | 0.91 | 0.005 | 0.91 | 0.80 | 1.04 |  | 0.180 |  |
| 2nd quartile | 1.00 |  |  |  | 1.00 |  |  |  |  |  |
| 3rd quartile | 1.27 | 1.03 | 1.56 | 0.024 | 1.11 | 0.96 | 1.27 |  | 0.161 |  |
| 4th quartile | 2.19 | 1.76 | 2.72 | <.0001 | 1.19 | 1.01 | 1.40 |  | 0.037 |  |
| **waist circumference** |  |  |  |  |  |  |  |  |  |  |
| 1st quartile | 0.54 | 0.40 | 0.73 | <.0001 | 0.95 | 0.82 | 1.11 |  | 0.506 |  |
| 2nd quartile | 1.00 |  |  |  | 1.00 |  |  |  |  |  |
| 3rd quartile | 1.30 | 1.04 | 1.63 | 0.021 | 1.07 | 0.93 | 1.22 |  | 0.345 |  |
| 4th quartile | 2.21 | 1.79 | 2.73 | <.0001 | 1.21 | 1.06 | 1.39 |  | 0.006 |  |
| **waist to hip ratio** |  |  |  |  |  |  |  |  |  |  |
| 1st quartile | 0.43 | 0.31 | 0.59 | <.0001 | 0.84 | 0.72 | 0.98 |  | 0.028 |  |
| 2nd quartile | 1.00 |  |  |  | 1.00 |  |  |  |  |  |
| 3rd quartile | 1.65 | 1.29 | 2.11 | <.0001 | 1.00 | 0.86 | 1.17 |  | 0.960 |  |
| 4th quartile | 3.22 | 2.50 | 4.15 | <.0001 | 1.19 | 1.02 | 1.40 |  | 0.027 |  |
| **waist to height ratio** |  |  |  |  |  |  |  |  |  |  |
| 1st quartile | 0.60 | 0.44 | 0.81 | <.0001 | 0.93 | 0.80 | 1.07 |  | 0.292 |  |
| 2nd quartile | 1.00 |  |  |  | 1.00 |  |  |  |  |  |
| 3rd quartile | 1.41 | 1.12 | 1.77 | 0.003 | 1.07 | 0.93 | 1.22 |  | 0.348 |  |
| 4th quartile | 2.65 | 2.15 | 3.26 | <.0001 | 1.29 | 1.13 | 1.48 |  | <.0001 |  |

Values are adjusted for sex, survey, education level, alcohol intake, smoking status, physical activity and time/smoking status interaction.
