# Supplementary material for: New indexes of body fat distribution and sex-specific risk of total and cause-specific mortality: a prospective cohort study
Source: BMC Public Health. 2018 Apr 2;18:427. doi: 10.1186/s12889-018-5350-8 (PMC5879745; doi:10.1186/s12889-018-5350-8)
Supplement: Supplementary file 3 — Different anthropometric measures and CVD mortality. Hazard ratios (HR) and confidence intervals (CI) for the association between the different anthropometric measures and CVD mortality for persons <=/> 60 years by quartiles; the second quartile was set as the reference category. (DOCX 21 kb) [file 12889_2018_5350_MOESM3_ESM.docx]

Additional file 3: Different anthropometric measures and CVD mortality

Table Hazard ratios (HR) and confidence intervals (CI) for the association between the different anthropometric measures and CVD mortality for persons <=/> 60 years by quartiles; the second quartile was set as the reference category.

q

| **CVD mortality** |  | **<= 60 years** |  |  |  |  | **>60 years** |  |  |  |
| --- | --- | --- | --- | --- | --- | --- | --- | --- | --- | --- |
|  | HR | 95% CI | | p-value | HR | 95% CI | |  | p-value |  |
| **Body mass index** |  |  |  |  |  |  |  |  |  |  |
| 1st quartile | 0.81 | 0.53 | 1.25 | 0.336 | 0.96 | 0.78 | 1.18 |  | 0.692 |  |
| 2nd quartile | 1.00 |  |  |  | 1.00 |  |  |  |  |  |
| 3rd quartile | 1.00 | 0.69 | 1.45 | 0.995 | 1.07 | 0.88 | 1.31 |  | 0.495 |  |
| 4th quartile | 1.89 | 1.35 | 2.64 | <.0001 | 1.20 | 0.98 | 1.47 |  | 0.078 |  |
| **Body adiposity index** |  |  |  |  |  |  |  |  |  |  |
| 1st quartile | 0.61 | 0.41 | 0.91 | 0.016 | 0.78 | 0.64 | 0.95 |  | 0.013 |  |
| 2nd quartile | 1.00 |  |  |  | 1.00 |  |  |  |  |  |
| 3rd quartile | 1.36 | 0.96 | 1.92 | 0.084 | 1.08 | 0.88 | 1.32 |  | 0.461 |  |
| 4th quartile | 2.53 | 1.75 | 3.64 | <.0001 | 1.29 | 1.02 | 1.63 |  | 0.037 |  |
| **waist circumference** |  |  |  |  |  |  |  |  |  |  |
| 1st quartile | 0.76 | 0.46 | 1.25 | 0.285 | 0.75 | 0.59 | 0.94 |  | 0.012 |  |
| 2nd quartile | 1.00 |  |  |  | 1.00 |  |  |  |  |  |
| 3rd quartile | 1.50 | 1.00 | 2.26 | 0.051 | 1.03 | 0.84 | 1.25 |  | 0.801 |  |
| 4th quartile | 2.92 | 2.00 | 4.26 | <.0001 | 1.26 | 1.04 | 1.54 |  | 0.021 |  |
| **waist to hip ratio** |  |  |  |  |  |  |  |  |  |  |
| 1st quartile | 0.52 | 0.30 | 0.90 | 0.019 | 0.80 | 0.64 | 1.01 |  | 0.062 |  |
| 2nd quartile | 1.00 |  |  |  | 1.00 |  |  |  |  |  |
| 3rd quartile | 2.06 | 1.33 | 3.18 | 0.001 | 1.01 | 0.81 | 1.25 |  | 0.938 |  |
| 4th quartile | 4.63 | 2.96 | 7.24 | <.0001 | 1.18 | 0.93 | 1.48 |  | 0.168 |  |
| **waist to height ratio** |  |  |  |  |  |  |  |  |  |  |
| 1st quartile | 0.76 | 0.44 | 1.29 | 0.307 | 0.79 | 0.64 | 0.98 |  | 0.033 |  |
| 2nd quartile | 1.00 |  |  |  | 1.00 |  |  |  |  |  |
| 3rd quartile | 1.85 | 1.22 | 2.80 | 0.004 | 1.05 | 0.86 | 1.28 |  | 0.621 |  |
| 4th quartile | 3.56 | 2.42 | 5.23 | <.0001 | 1.23 | 1.01 | 1.50 |  | 0.035 |  |

Values are adjusted for sex, survey, education level, alcohol intake, smoking status, physical activity and time/smoking status interaction.
