# Supplementary material for: New indexes of body fat distribution and sex-specific risk of total and cause-specific mortality: a prospective cohort study
Source: BMC Public Health. 2018 Apr 2;18:427. doi: 10.1186/s12889-018-5350-8 (PMC5879745; doi:10.1186/s12889-018-5350-8)
Supplement: Supplementary file 4 — Different anthropometric measures and cancer mortality. Hazard ratios (HR) and confidence intervals (CI) for the association between the different anthropometric measures and cancer mortality for persons <=/> 60 years by quartiles; the second quartile was set as the reference category. (DOCX 21 kb) [file 12889_2018_5350_MOESM4_ESM.docx]

Additional file 4: Different anthropometric measures and cancer mortality

Table Hazard ratios (HR) and confidence intervals (CI) for the association between the different anthropometric measures and cancer mortality for persons <=/> 60 years by quartiles; the second quartile was set as the reference category.

q

| **Cancer mortality** |  | **<= 60 years** |  |  |  |  | **>60 years** |  |  |  |
| --- | --- | --- | --- | --- | --- | --- | --- | --- | --- | --- |
|  | HR | 95% CI | | p-value | HR | 95% CI | |  | p-value |  |
| **Body mass index** |  |  |  |  |  |  |  |  |  |  |
| 1st quartile | 0.61 | 0.42 | 0.89 | 0.010 | 1.22 | 0.95 | 1.57 |  | 0.112 |  |
| 2nd quartile | 1.00 |  |  |  | 1.00 |  |  |  |  |  |
| 3rd quartile | 0.87 | 0.64 | 1.20 | 0.409 | 1.03 | 0.80 | 1.34 |  | 0.808 |  |
| 4th quartile | 1.21 | 0.89 | 1.63 | 0.226 | 1.42 | 1.10 | 1.83 |  | 0.007 |  |
| **Body adiposity index** |  |  |  |  |  |  |  |  |  |  |
| 1st quartile | 0.86 | 0.61 | 1.20 | 0.362 | 1.03 | 0.81 | 1.31 |  | 0.824 |  |
| 2nd quartile | 1.00 |  |  |  | 1.00 |  |  |  |  |  |
| 3rd quartile | 1.12 | 0.80 | 1.56 | 0.516 | 1.13 | 0.87 | 1.47 |  | 0.364 |  |
| 4th quartile | 1.89 | 1.33 | 2.68 | <.0001 | 1.21 | 0.90 | 1.64 |  | 0.213 |  |
| **waist circumference** |  |  |  |  |  |  |  |  |  |  |
| 1st quartile | 0.53 | 0.34 | 0.81 | 0.004 | 1.06 | 0.80 | 1.40 |  | 0.691 |  |
| 2nd quartile | 1.00 |  |  |  | 1.00 |  |  |  |  |  |
| 3rd quartile | 1.29 | 0.91 | 1.82 | 0.150 | 1.20 | 0.93 | 1.54 |  | 0.153 |  |
| 4th quartile | 1.98 | 1.43 | 2.75 | <.0001 | 1.20 | 0.93 | 1.56 |  | 0.169 |  |
| **waist to hip ratio** |  |  |  |  |  |  |  |  |  |  |
| 1st quartile | 0.46 | 0.30 | 0.72 | 0.001 | 0.87 | 0.66 | 1.15 |  | 0.333 |  |
| 2nd quartile | 1.00 |  |  |  | 1.00 |  |  |  |  |  |
| 3rd quartile | 1.26 | 0.87 | 1.83 | 0.225 | 0.99 | 0.75 | 1.30 |  | 0.925 |  |
| 4th quartile | 2.30 | 1.56 | 3.38 | <.0001 | 1.18 | 0.88 | 1.57 |  | 0.269 |  |
| **waist to height ratio** |  |  |  |  |  |  |  |  |  |  |
| 1st quartile | 0.46 | 0.30 | 0.72 | 0.001 | 1.06 | 0.82 | 1.38 |  | 0.654 |  |
| 2nd quartile | 1.00 |  |  |  | 1.00 |  |  |  |  |  |
| 3rd quartile | 1.11 | 0.79 | 1.55 | 0.552 | 1.18 | 0.92 | 1.52 |  | 0.196 |  |
| 4th quartile | 1.93 | 1.42 | 2.63 | <.0001 | 1.44 | 1.12 | 1.85 |  | 0.004 |  |

**Values are adjusted for sex, survey, education level, alcohol intake, smoking status, physical activity and time/smoking status interaction**
